# A Methodological Approach for Evaluating the Genotypic Variation for Physiological Adaptation of Potato Wild Relatives for Heat Tolerance Breeding

**DOI:** 10.3390/plants14193096

**Published:** 2025-10-08

**Authors:** Ikram Bashir, Rodrigo Nicolao, Eduardo Pereira Shimoia, Luciano do Amarante, Caroline Marques Castro, Gustavo Heiden

**Affiliations:** 1 Laboratório de Botânica, Universidade do Vale do Taquari (Univates), Lajeado 95914-014, RS, Brazil; 2 Programa de Pós-Graduação em Agronomia, Faculdade de Agronomia Eliseu Maciel, Universidade Federal de Pelotas (UFPel), Pelotas 96010-900, RS, Brazil; 3 Programa de Pós-Graduação em Fisiologia Vegetal, Universidade Federal de Pelotas (UFPel), Pelotas 96160-000, RS, Brazil; 4 Embrapa Clima Temperado, P.O. Box 403, Pelotas 96010-971, RS, Brazil

**Keywords:** wild potatoes, heat stress, genetic variability, genotypic values, heat tolerance coefficient, multivariate analysis

## Abstract

Wild potato relatives are vital for breeding programs to tackle rising temperatures. This study proposes a methodological approach based on the examination of genetic variation among 19 accessions belonging to *Solanum chacoense* and *Solanum commersonii* from the Embrapa Potato Genebank under heat stress (HS). Heat tolerance coefficient (HTC) was calculated using genotypic values predicted through mixed models. After 15 days of heat stress (DHS), a significant variation in gas exchange and chlorophyll fluorescence indicates strong breeding potential and photosystem resilience. By 35 DHS, increased pigment variation suggests acclimation. Based on predicted genotypic values, *S. chacoense* outperforms *S. commersonii* in tuber production and gas exchange under HS, and principal component analysis (PCA) performed using the HTC shows early resistance driven by photosynthesis, mid-term by tuber yield, and long-term by gas exchange and tuber production. Genotypes BRA00167017-3, BRA00167023-1, BRA00167025-6, and BRA00167028-0 excel in heat comprehensive evaluation values (HCEVs)/comprehensive principal component value (F) rankings, demonstrating robust photosynthesis, thermoregulation, and tuber yield. Cluster analysis identifies these as highly tolerant, ideal for breeding heat-resilient potatoes. These PCA-derived weights and genotype clustering system provide a precise tool for selecting heat-tolerant wild potato germplasm, categorizing them into highly tolerant, moderately tolerant, sensitive with late recovery, and highly sensitive groups acquired for specific objectives of the breeding programs to climate change.

## 1. Introduction

The detrimental effects of heat stress (HS) on potato cultivation are multifaceted, impacting both yield quantity and nutritional quality. Elevated temperatures accelerate leaf senescence, diminish photosynthetic rates, and impair assimilate translocation to developing tubers, often resulting in smaller, fewer, or malformed tubers [1]. Studies have shown that for every 1 °C increase above the optimal temperature, tuber yield can decline by 5–10%, with even greater losses in tropical and subtropical regions where potatoes are increasingly cultivated [2]. Beyond yield, HS compromises starch content and increases glycoalkaloid levels, posing risks to food safety and marketability [3]. These impacts reverberate through food security frameworks, particularly in developing nations where potatoes serve as a primary calorie source and a buffer against cereal crop failures [4]. The International Potato Center (CIP) estimates that without adaptive measures, HS could reduce global potato production by up to 25% by 2050, exacerbating hunger and economic instability in vulnerable regions [5]. Thus, enhancing the resilience of potato crops to HS is not merely an agricultural priority but a critical step toward safeguarding livelihoods and nutritional stability.

Wild potato relatives (*Solanum* spp.), numbering over 100 species, offer a rich genetic reservoir for addressing these challenges [6]. Unlike cultivated varieties, which have undergone extensive domestication and genetic narrowing, wild species exhibit a broader range of adaptability to abiotic stresses, including heat, drought, and salinity [7,8]. For instance, species such as *Solanum chacoense* and *Solanum acaule*, native to arid and high-altitude environments, possess traits like enhanced thermotolerance and efficient water use that are absent or diminished in modern cultivars [9]. Introgressing these traits into cultivated potatoes through breeding programs has shown promise in preliminary trials, with wild-derived lines under stress conditions [10]. However, the successful utilization of wild germplasm requires overcoming barriers such as crossability issues, linkage drag, and the complexity of polygenic stress tolerance traits [11]. Advances in marker-assisted selection and genomic tools have begun to bridge these gaps, enabling breeders to pinpoint and transfer desirable alleles with greater precision [12].

Central to this breeding effort is the identification and deployment of reliable selection parameters, specifically physiological and morphological traits that confer heat tolerance. Physiological traits, such as photosynthetic efficiency, chlorophyll stability, and membrane thermostability, are robust indicators of a plant’s ability to maintain metabolic function under high temperatures [13]. For example, genotypes able to maintain higher photosynthetic rates under HS are able to divert photoassimilate supplies to tubers, which is a key physiological response to ensure higher yield under stress [14]. Similarly, morphological traits like leaf thickness, stomatal density, and root architecture influence heat dissipation and water uptake, critical for coping with thermal and concurrent drought stress [15]. Field studies have demonstrated that potato lines with thicker leaves and deeper root systems exhibit reduced canopy temperatures and improved tuberization under HS, highlighting the practical utility of these traits [16,17,18,19]. By integrating these parameters into screening protocols, breeders can systematically evaluate and select genotypes with enhanced adaptability, moving beyond traditional reliance on yield alone.

To navigate the complexity of trait interactions and environmental variability, multivariate analysis has emerged as a powerful tool in modern plant breeding. Techniques such as principal component analysis (PCA) and canonical discriminant analysis allow researchers to dissect the relationships among multiple traits, identifying those most strongly associated with heat tolerance [20]. However, traditional statistical methods often falter in accounting for genotype-by-environment (G × E) interactions, a critical factor in HS studies where performance varies across locations and seasons [21]. Mixed model methodologies, particularly best linear unbiased prediction (BLUP), address this limitation by providing a framework to estimate genotypic effects while adjusting for random environmental and experimental noise [22]. BLUP values enhance selection accuracy by predicting breeding values for traits like tuber yield or physiological stability, enabling the identification of superior genotypes across diverse conditions [23,24]. In potato breeding, BLUP has been successfully applied to evaluate wild-derived populations, revealing stable performers under HS that might be overlooked by conventional means [25]. Coupling BLUP with genomic selection further accelerates progress, linking phenotypic data to underlying genetic markers for rapid trait introgression [26].

This study aims to harness wild potato diversity to develop heat-tolerant cultivars, addressing the potential threat of HS to potato cultivation and global food security. By employing physiological and morphological traits as selection criteria, leveraging multivariate analysis with mixed models like BLUP, and studying the genetics of these traits under HS, we seek to identify and validate tolerant genotypes suitable for breeding programs. The integration of these approaches offers a comprehensive strategy to enhance potato resilience, ensuring sustained productivity in a warming world. As climate change intensifies, such efforts are vital to securing the future of this indispensable crop and the communities that depend on it. Thus, we hypothesize that there is substantial genotypic heterogeneity across wild potato accessions in how they respond to HS in terms of gas exchange, pigment, and chlorophyll fluorescence traits, as well as morphological traits, and that accessions that are particularly heat-tolerant can be selected based on these characteristics.

## 2. Results

### 2.1. Genetic and Environmental Influences on Potato Traits Under Contrasting Conditions

**Figure 1:**
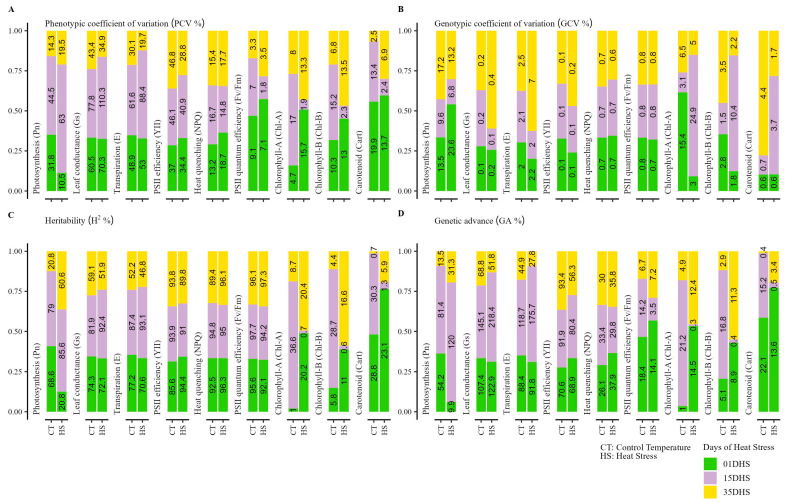
Comparative analysis of (**A**) phenotypic coefficient of variation (PCV%), (**B**) genotypic coefficient of variation (GCV%), (**C**) heritability (H^2^%), and (**D**) genetic advance (GA%) for potato wild relatives (*Solanum chacoense* and *S. commersonii*, Solanaceae) physiological traits under control treatment (CT) and heat stress (HS) conditions after 1, 15, and 35 days of heat stress (DHS).

Figure 1A–D presents a detailed analysis of nine physiological traits: photosynthesis (Pn), leaf conductance (Gs), transpiration (E), PSII efficiency (YII), heat dissipation (NPQ), PSII quantum efficiency (Fv/Fm), chlorophyll-A (Chl-A), chlorophyll-B (Chl-B), and carotenoid (Cart) under control (CT) and heat stress (HS) conditions at 1, 15, and 35 days of heat stress (DHS), with genetic parameters including heritability (H^2^%), genetic advance (GA%), genotypic coefficient of variation (GCV%), and phenotypic coefficient of variation (PCV%). Pn showed a contrary response under CT and HS conditions; for example, initial heritability was high (69%) under CT and low (21%) under HS at 1 DHS, increasing to 61% by 35 DHS, with a peak genetic advance of 120% at 15 DHS under stress, while GCV and PCV were also high (44% and 63% at 15 DHS), indicating variability. Gs and E maintain high heritability (47–93%) under both conditions, respectively, from 1 DHS to 35 DHS, and a genetic advance along with PCV and GCV were also recorded as high, but for both traits, the highest values for GA, GCV, and PCV were observed at 15 DHS. PSII efficiency, heat dissipation, and the Fv/Fm ratio exhibit high heritability between 89 and 98%, and the GA percentages were higher for YII and NPQ; for Fv/Fm, a high GA was observed after 35 DHS. Chlorophyll fluorescence traits (YII, NPQ, and Fv/Fm) showed high, moderate, and low genotypic and phenotypic coefficients, respectively. The Fv/Fm ratio maintains near-perfect heritability (92–99%) but low to medium genetic advance (7–19%), with reduced GCV and PCV with growth cycle. Chlorophyll-A and -B and carotenoids showed low heritability at 01 and 35 DHS; however, medium heritability was observed under CT at 15 DHS for Chl-A and Cart. Under HS conditions, the genetic advance (GA%) was higher as compared with CT at 1 and 35 DHS for Chl-A and -B. For carotenoid content, GA was higher under CT. All the pigment traits (Chl-A, Chl-B, and Cart) showed higher levels of PCV as compared with the genetic factors (GCV). These results correlate strongly, as high heritability and genetic advance in early stress (1–15 DHS) for traits like leaf conductance and transpiration align with elevated GCV/PCV, suggesting adaptive variability for breeding, while prolonged stress (35 DHS) reduces these parameters for chlorophyll and carotenoids, likely due to oxidative damage. The interdependence of Pn, Gs, and E is evident in their correlated GCV/PCV increases, while chlorophyll and carotenoid declines suggest a linked degradation process, contrasting with the stability of PSII efficiency and Fv/Fm, which indicate inherent resilience. HS initially boosts diversity for selection, but long-term exposure erodes genetic CT, emphasizing the need to target early stress responses in breeding programs.

The results of the study reveal significant genetic and environmental influences on key potato traits under CT and HS conditions, as assessed through descriptive statistics, analysis of variance (ANOVA), and genetic parameters such as GCV, PCV, H^2^, and GA (Table 1). For fresh shoot weight, the H^2^ was moderate at >40%, with a genotypic coefficient of variation (GCV) of 22% and a phenotypic coefficient of variation (PCV) of >33% under CT and HS conditions, suggesting a substantial genetic contribution to phenotypic variation. Dry shoot weight (DSW) was less sensitive to environmental stress. The heritability was also moderate (48%) under HS, while low heritability was observed under CT conditions. However, low PCV, GCV, and genetic advance were observed under both conditions. Wild potato tuber traits demonstrate high heritability under both conditions, ranging from 60% to 90% with a high GCV and PCV percentage, although PCV was higher than the GCV, indicating a strong genetic basis and their importance in the potato breeding program (Table 1).

### 2.2. Heat Stress Affects the Physiological and Tuber Trait Genetic Variability

Among physiological traits, transpiration (E) and PSII efficiency (YII), in studied wild germplasm, showed a significant increase under HS conditions by 11% and 53%, respectively. NPQ and Fv/Fm were significantly lower by 4% and 2%, respectively (Figure S1). HS significantly affects the wild germplasm for the performance of morphological traits and shows a decline in FSW (17%), NT (30%), FTW (44%) and DM (14%). However, on average, Gs, Chl-A, Chl-B, and Cart showed a slight increase, and Pn and DSW were observed to slightly decrease under HS, but these changes were not statistically significant (Figure S1).

The statistical analysis along with the heat tolerance coefficient (HTC) of the number of tubers, tuber weight, dry matter, fresh shoot weight, dry shoot weight, photosynthesis, leaf conductance, transpiration, potential efficiency of PSII, heat quenching, Fv/Fm ratio, as well as chlorophyll-A and -B and the carotenoid content of 19 wild potato accessions is shown in Table 1 and Table 2. The heat tolerance coefficient (HTC) was calculated for all used variables from the BLUP values predicted by the mixed models. The HTC value varies among wild accessions for each indicator. The average HTC in leaf conductance, transpiration, PSII efficiency, chlorophyll-A and -B, and carotenoids was greater than 1.00 (Table 2), while morphological traits and Pn, NPQ, and Fv/Fm were less than 1.00. E had the highest HTC value.

Under HS treatment, the transpiration (E), YII, Chl-A and -B, and Cart increased significantly (Table 2). HS leads to decreases in the FSW, and all tuber-related traits showed no significant differences when compared with the CT group (Table 1). However, the indicators in all accessions did not follow the same trend; for example, 2 out of 19 accessions in number of tubers (Table S3), 4 accessions in the dry matter content (Table S3), 8 accessions in photosynthesis, and 3 accessions in the maximum efficiency of PSII increased under HS compared with CT.

The results demonstrate significant genotypic variation among wild potato accessions in response to HS across multiple physiological and biochemical traits. At 1 day of heat stress, genotype (G) had a highly significant (*p* < 0.001) effect on photosynthetic rate (Pn), leaf conductance (Gs), transpiration (E), chlorophyll fluorescence traits (YII, NPQ, Fv/Fm), and carotenoid content (Cart), but not on chlorophyll-A (Chl-A), chlorophyll-B (Chl-B), or carotenoid (Cart). Treatment (T) effects were significant only for YII and Fv/Fm, while genotype × treatment (G × T) interactions were significant for Pn, Gs, E, YII, NPQ, Fv/Fm, and Cart, indicating differential stress responses among accessions (Table S2). By 15 days after stress, genotypic effects remained highly significant for all traits except Chl-B, while treatment effects became more pronounced, particularly for Pn, Gs, NPQ, and Fv/Fm. The G × T interaction was significant for most traits, confirming that wild potato accessions varied in their HS adaptation over time (Table S2). At 35 days, treatment effects intensified, with HS significantly reducing Pn, Gs, E, YII, and NPQ while increasing Chl-a, Chl-b, and Cart, suggesting potential compensatory mechanisms. The G × T interaction remained significant for YII, NPQ, and Fv/Fm, though less pronounced for other traits. For morphological traits (FSW, DSW, FTW, DMC), genotypes exhibited strong variation, while HS significantly reduced fresh shoot weight (FSW) and tuber weight (FTW) but increased dry matter content (DM). The G × T interaction was only significant for DM, indicating that genotypic differences in adaptation may influence heat responses. These findings highlight substantial genetic diversity in wild potato accessions under HS, with potential candidates for breeding heat-tolerant varieties (Table S2).

The physiological responses were observed by predicting true genotypic values, or BLUP values, under CT and HS conditions, which were calculated from observations at 1, 15, and 35 DHS. Thereafter, BLUP values were used to estimate the heat tolerance coefficient (HTC) mean for the observed traits (Table 2). HTC values of < 1 suggest sensitivity, and >1 indicate enhanced performance under stress of the respective traits. The results highlight temporal dynamics, with initial stress impacts, followed by acclimation by 35 DHS.

Photosynthetic rates were similar at 1 DHS between CT (11.16 ± 2.7), with BLUP values ranging from 5.24 to 15.91, and HS (10.75 ± 1.4), with BLUP values of 8.29 to 13.18, with no significant difference (*p* > 0.05) and a HTC of 1.02, indicating minimal initial stress impact. By 15 DHS, HS reduced photosynthesis (6.29 ± 3.6, BLUP: 1.23–12.40) compared with CT (8.49 ± 3.5, BLUP: 1.94–13.12), with no significant difference except a HTC of 0.77, reflecting sensitivity. At 35 DHS, HS plants showed significantly higher photosynthesis (10.24 ± 1.3, BLUP: 7.82–13.23) than CT (7.84 ± 1.1, BLUP: 5.56–10), with a HTC of 1.32, suggesting adaptation and enhanced photosynthetic capacity under prolonged stress (Table 2).

Leaf conductance at 1 DHS was comparable between CT (0.12 ± 0.1, BLUP: 0.02–0.29) and HS (0.13 ± 0.1, BLUP: 0.03–0.33), with no significant difference and a HTC of 1.15. At 15 DHS, HS significantly reduced conductance (0.08 ± 0.1, BLUP: 0.01–0.28) compared with CT (0.16 ± 0.1, BLUP: 0.02–0.39), with a HTC of 0.60, indicating stomatal closure. By 35 DHS, HS significantly increased conductance (0.27 ± 0.1, BLUP: 0.15–0.47) compared with CT (0.14 ± 0.1, BLUP: 0.06–0.27), with a HTC of 2.01, reflecting improved stomatal regulation (Table 2).

Transpiration was lower at 1 DHS, but there were no significant differences in average responses under CT (1.78 ± 0.8, BLUP: 0.45–3.4) and HS (1.88 ± 0.9, BLUP: 0.46–3.62), with a HTC of 1.09. At 15 DHS, no significant difference was observed (*p* > 0.05) between HS (1.88 ± 1.6, BLUP: 0.29–5.45) and CT (2.0 ± 1.2, BLUP: 0.3–3.92), with a HTC of 1.06, though HS showed higher variability (CV% 83.26%). By 35 DHS, HS significantly increased transpiration (4.77 ± 0.7, BLUP: 3.44–6.42) compared with CT (1.8 ± 0.5, BLUP: 0.87–2.64), with a HTC of 2.77, likely driven by increased conductance (Table 2).

PSII efficiency and heat dissipation showed no significant differences (*p* > 0.05) for the BLUP values predicted at 1 DHS and 15 DHS between CT and HS, with a HTC of 1.02 and 1.03, respectively. By 35 DHS, HS significantly increased PSII efficiency (0.18 ± 0.1, BLUP: 0.08–0.28) compared with CT (0.12 ± 0.1, BLUP: 0.02–0.19) (** *p* ≤ 0.01), with a HTC of 2.42, indicating enhanced photochemical efficiency, while HS significantly (** *p* ≤ 0.01) reduced heat dissipation (0.59 ± 0.1, BLUP: 0.36–0.74) compared with CT (0.69 ± 0.1, BLUP: 0.5–0.91), with a HTC of 0.86, suggesting reduced non-photochemical quenching under prolonged stress (Table 2).

The Fv/Fm ratio significantly (* *p* ≤ 0.05) decreased under HS (0.70 ± 0.1, BLUP: 0.59–0.78) at 1 DHS compared with CT (0.75 ± 0.1, BLUP: 0.5–0.79), with a HTC of 0.95, indicating minor stress on PSII. At 15 and 35 DHS, no significant difference was observed between CT vs. HS (0.75 ± 0.1 vs. 0.76 ± 0.01 and 0.76 ± 0.02 vs. 0.75 ± 0.03 were similar (*p* > 0.05)), with a HTC of 1.02 vs. 0.99 at 15 and 35 DHS, respectively, indicating stable PSII function (Table 2).

At 1 DHS, HS significantly reduced chlorophyll-A and B compared with CT, with a HTC of 0.90. At 15 DHS, HS increased chlorophyll-A (2.05 ± 0.2, BLUP: 1.79–2.35), while there was no significant difference for chlorophyll-B under CT vs. HS conditions, with a HTC of >1. At 35 DHS, HS increased chlorophyll-A and -B as compared with CT; however, under HS at 35 DHS, Chl-A and -B were higher as compared with 01 and 15 DHS, indicating pigment accumulation under stress (Table 2).

At 1 DHS, for carotenoid content (Cart), no significant difference (*p* > 0.05) was observed between CT and HS, with a HTC of 0.98. At 15 DHS, HS increased carotenoid content (0.42 ± 0.03, BLUP: 0.37–0.47) compared with CT (0.38 ± 0.03, BLUP: 0.33–0.43), with a HTC of 1.11. At 35 DHS, HS further increased carotenoids (0.42 ± 0.01, BLUP: 0.4–0.44) compared with CT (0.38 ± 0.01, BLUP: 0.36–0.4) (*** *p* ≤ 0.001), with a HTC of 1.09, indicating enhanced photoprotection (Table 2).

Overall, the results demonstrate an initial negative impact of HS on photosynthesis, leaf conductance, and pigment content at 1 and 15 DHS, followed by significant acclimation by 35 DHS, with HS plants exhibiting higher photosynthesis, conductance, transpiration, PSII efficiency, and pigment levels, supported by HTC values of > 1, indicating adaptive responses to prolonged HS.

**Figure 2:**
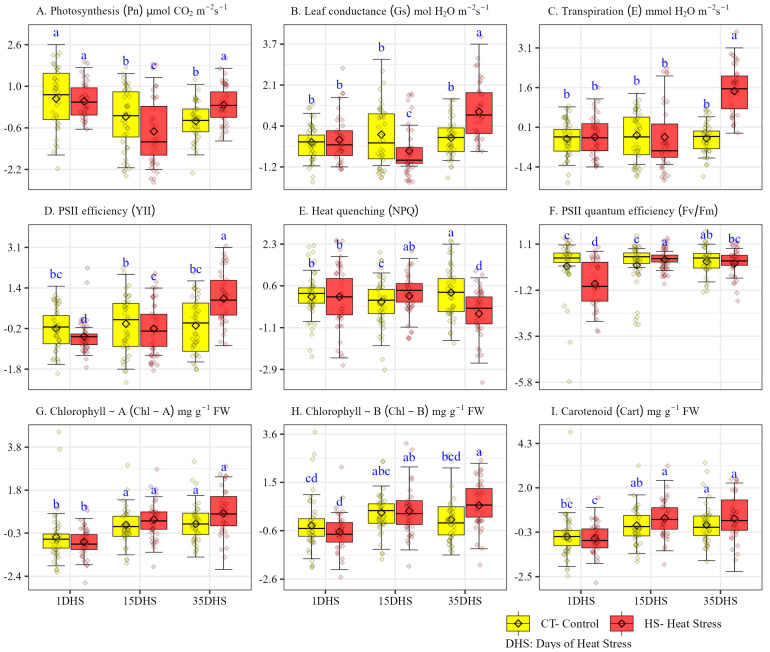
Average temporal responses of physiological traits in potato wild relatives (*Solanum chacoense* and *S. commersonii*, Solanaceae) after 1 day of heat stress (DHS), 15 DHS, and 35 DHS application. Small letters indicate the Tukey test mean differences.

In terms of temporal average responses of wild potato genotypes calculated with growth chamber (Figure 2), the Pn was lower at 15 DHS (7.39) and highest at 1 DHS (10.96), although Pn under HS decreased at 1 DHS and 15 DHS while increasing at 35 DHS by 3.59%, 25.92%, and 30.71%, respectively. Pn of wild genotypes measured at 15 DHS and 35 DHS showed a significant difference in performance under CT and HS conditions. The Gs of genotypes was significantly higher at 35 DHS by 0.206 as compared with 1 DHS and 15 DHS, which showed an average of 0.13 and 0.12, respectively. Higher Gs values were observed at the start of the HS period (1 DHS) and at a prolonged period (35 DHS). The significant difference in Gs values under CT and HS conditions were observed at 15 DHS and 35 DHS. An increase was observed in transpiration rate for the used germplasm at HS condition compared with CT, but the only significant increase was at 35 DHS. Under CT, the highest average E observed was at 15 DHS and under HS at 35 DHS by 1.99 and 4.77, respectively. CF trait, operating PSII efficiency (YII) observed higher values at 35 DHS, followed by 15 DHS and 1 DHS with observed values 0.146, 0.116, and 0.101, respectively. At 35 DHS, YII was observed to increase significantly under HS by 53.89% as compared with CT conditions. On the contrary, NPQ values were significantly lower at 35 DHS by 14.73%. There were no significant differences in the values of the NPQ when compared at 1 DHS, 15 DHS, and 35 DHS and ranged from 0.387 to 0.909 under CT conditions and 0.328 to 0.861 under HS conditions. Fv/Fm values were higher significantly at 1 DHS while there were no significant differences observed at 15 DHS and 35 DHS. In pigment analysis, the highest value for chlorophyll-A and -B and carotenoid (Cart) were observed at 35 DHS, and there was a significant increase in chlorophyll under HS and a statistically non-significant increase observed for Cart. Low Chl-A and -B and Cart were observed at early stages (01 DHS), and there was also no significant difference in the performance of this trait at this stage. Cart values were significantly higher under HS with an increase of 10.85% at 15 DHS.

### 2.3. Physiological Trait Association with Heat Stress

**Figure 3:**
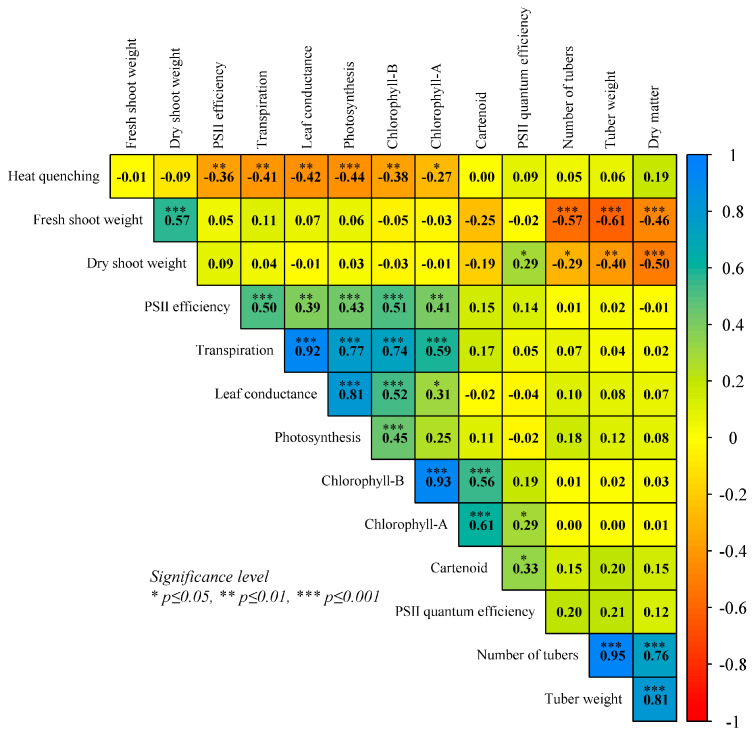
Correlation analysis of HTC values of measured traits in wild potato accession of (*Solanum chacoense* and *S. commersonii*, Solanaceae) after 1, 15, and 35 days of heat stress (DHS).

The Pearson correlation was performed by using HTC values, among all measured indicators over three time periods evaluated at 1, 15, and 35 DHS is presented in Figure 3. The tuber yields related traits such as NT, TW, and DM positively and significantly correlated with each other, indicating a core physiological process; however, resources are diverted away from vegetative growth, explaining the moderate to low significant negative correlation with fresh and dry shoot weight. Heat quenching (NPQ) showed a significant negative correlation (−0.27 to −0.44) with most of the traits such as YII, E, Gs, Pn, Chl-B, and Chl-A, which showed potato genotypes adapting to closing stomata and activating the photoprotection mechanism to save it from further damage. But this adaptation comes at the cost of reducing Pn and E, leading to hindered tuber yield. Gas exchange traits (Pn, Gs, and E) had high correlations (0.77–0.92) among them, which is the natural response physiology of the potato plant essential for tuber growth. Pigments traits (Chl-A, Chl-B, and Cart), also observed to have significant positive correlations (0.56–0.93), reflect the importance of capturing light and photoprotection to maintain the photosynthetic output. PSII efficiency (YII) showed a moderate positive correlation (0.39–0.51) with E, Gs, Pn, Chl-B, and Chl-A, indicating the health of the photosynthetic machinery. PSII quantum efficiency (Fv/Fm) was observed to have weak association with DSW, Chl-A and Cart. Among pigment traits, Chl-A and Chl-B showed a moderate positive correlation with YII, Gs, and Pn.

### 2.4. Wild Potato Genotypes Acclimatize to High-Temperature Stress at Tuberization Stage

**Figure 4:**
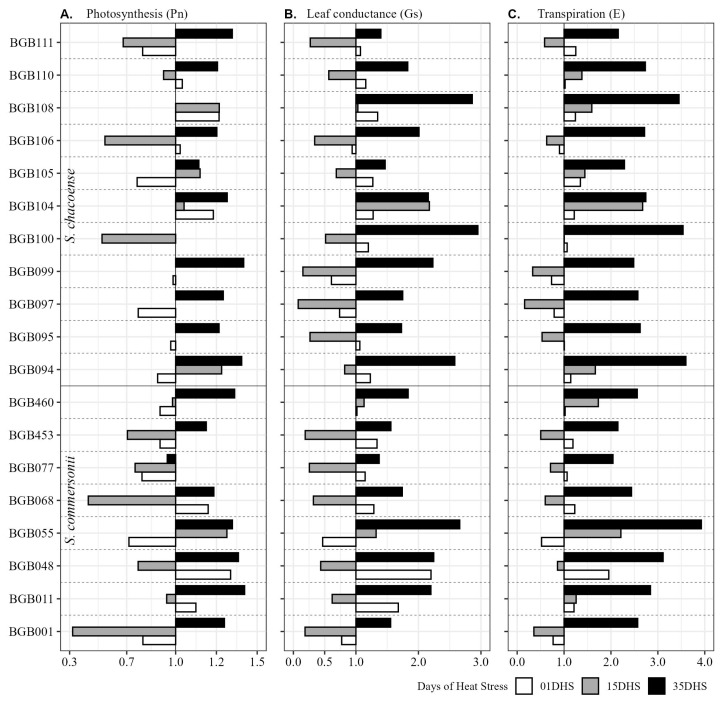
Wild potato genotypes of *S. chacoense* and *S. commersonii* from Embrapa Potato Genebank performance as heat tolerance coefficient under control temperature (CT) and heat stress (HS) conditions after 1, 15 and 35 DHS, for photosynthesis (Pn), leaf conductance (Gs), and transpiration (E).

**Figure 5:**
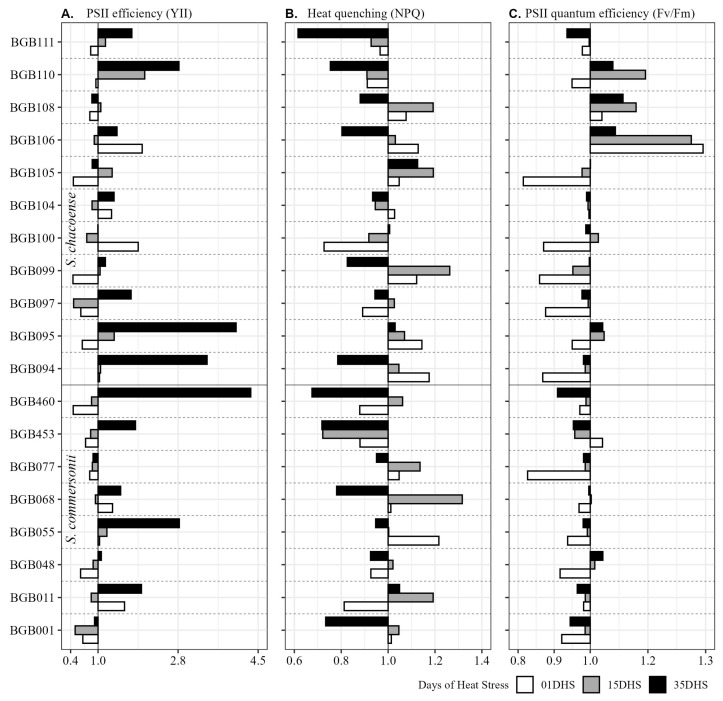
Wild potato genotypes of *S. chacoense* and *S. commersonii* from Embrapa Potato Genebank performance as heat tolerance coefficient under control temperature (CT) and heat stress (HS) conditions after 1, 15 and 35 DHS, for PSII efficiency (YII), heat quenching (NPQ), and PSII quantum efficiency (Fv/Fm).

Figure 4 and Figure 5 represent the heat tolerance coefficient (HTC) values for 19 genotypes of two wild potato species (*S. commersonii* and *S. chacoense*) across physiological variables measured at three timepoints (1, 15, and 35 days of heat stress) under HS conditions relative to CT conditions. Figure S2 represents the HTC values of 19 wild potato genotypes for morphological traits. HTC values of >1 indicate higher performance under stress, while values <1 suggest reduced performance.

For all the screened wild potato germplasm, pigment levels (Chl-A, Chl-B, and Cart) are maintained, supporting photosynthetic activity. In this study, 16 out of 19 wild potato accessions were tuberized by simulating natural heat temperatures under the climate chamber. Four of the accessions, BGB001, BGB055, BGB099, and BGB453, did not produce any tuber under both conditions. BGB460 produces tubers under CT conditions only (Table S3).

In terms of group responses, we divided the screened germplasm into four categories based on their responses to HS. Highly Tolerant (BGB011, BGB048, BGB055, BGB094, BGB100, BGB104, BGB108, and BGB110) because of these genotypes’ ability of exceptional recovery or even enhancement of the Pn, Gs, and E by 35 DHS (Figure 4). These genotypes also demonstrate stable or improved YII and Fv/Fm under long-term heat stress, indicating no permanent damage to photosynthetic apparatus by effective, stable, and efficient heat quenching (NPQ) (Figure 5B).

Moderately tolerant genotypes (BGB068, BGB077, BGB095, BGB097, BGB099, BGB105, BGB106, BGB111, and BGB460) show balanced responses, with some early reductions in Pn, Gs, E, and YII but strong recovery by 35 DHS. Genotypes showed a functional photoprotection mechanism against excessive heat (Figure 5B)

Sensitive genotypes (BGB001 and BGB453) exhibit early stress sensitivity or reduction in Pn, Gs, and E (Figure 4) but stabilize or recover by 35 DHS. These genotypes demonstrate significant early decline in YII, which indicates photoinhibition and less ability to safely dissipate excessive energy (NPQ), and recovery was also low or minimal (Figure 5).

On specie levels, *S. commersonii* shows initial sensitivity to HS, with reduced photosynthesis, leaf conductance, and transpiration early on, likely due to stomatal closure and photoinhibition. By 35 days of heat stress (DHS), most genotypes recover, enhancing photosynthetic rates and gas exchange, indicating acclimation through improved stomatal function and enzyme stability. PSII efficiency varies, with some genotypes recovering robustly, while NPQ and Fv/Fm remain stable, reflecting effective photoprotection. Pigment levels (chlorophylls and carotenoids) are generally maintained or slightly enhanced, supporting photosynthesis. Agronomically, shoot biomass is moderately reduced, but tuber production is often severely impacted, suggesting limited reproductive resilience under HS.

*Solanum chacoense* exhibits greater overall heat tolerance than *S. commersonii*. Photosynthesis and gas exchange (leaf conductance and transpiration) show early variability but significantly improved by 35 DHS, reflecting strong thermoregulation and carbon assimilation. PSII efficiency is generally stable or enhanced, indicating a resilient photosynthetic apparatus. NPQ and Fv/Fm are consistently stable, ensuring effective photoprotection. Pigment levels are well-maintained, supporting sustained photosynthesis. Agronomically, tuber traits show better resilience compared with *S. commersonii*, with moderate shoot biomass reductions, suggesting a balanced vegetative and reproductive response to HS.

### 2.5. Principal Components Analysis

The principal component analysis (PCA) results for wild potato genotypes under HS at 1, 15, and 35 days of heat stress (DHS) reveal key physiological and agronomic trait variations, providing insights into their heat tolerance and implications for breeding heat-resilient potatoes (Table 3). The PCA identifies major components (PC1–PC5) explaining the variance in traits. At 1 DHS, the first principal component (F1, 32.95% variance) is strongly associated with Pn, pigment content (Chl-A, Chl-B, Cart), and tuber traits (NT, FTW, DMC), indicating that early HS responses are dominated by photosynthetic activity and reproductive output. Genotypes with high PC1 scores maintain photosynthetic capacity and tuber initiation despite heat, suggesting early tolerance. PC2 (20.13%) is driven by leaf conductance and transpiration, reflecting the importance of gas exchange and thermoregulation in early stress. PC3 (15.55%) correlates with PSII efficiency and Fv/Fm, highlighting photosystem health as a key factor. The negative loading of shoot biomass (FSW, DSW) on PC1 suggests a trade-off, where genotypes prioritizing photosynthesis and tuber traits may reduce vegetative growth. For breeding, selecting genotypes with high PC1 and PC2 scores would prioritize early photosynthetic stability and gas exchange, critical for maintaining yield under short-term HS.

At 15 DHS, PC1 (26.69% variance) is strongly linked to agronomic traits (FSW, NT, FTW, DM), indicating that mid-term HS responses shift toward biomass and tuber production. Genotypes with high PC1 scores maintain tuber yield and shoot growth, suggesting resilience in reproductive and vegetative development. PC2 (21.11%) is dominated by photosynthesis, leaf conductance, and transpiration, reflecting the continued importance of gas exchange and carbon assimilation for acclimation. PC3 (19.04%) is driven by pigment content (Chlorophyll-A and -B, carotenoids), indicating that pigment stability supports photosynthetic recovery. PC4 (11.42%) correlates with Fv/Fm and PSII efficiency, emphasizing photosystem recovery. The negative loading of shoot biomass on PC1 suggests that some genotypes prioritize tuber production over vegetative growth. For breeding, genotypes with balanced PC1 and PC2 scores are ideal, as they combine sustained tuber yield with photosynthetic and gas exchange resilience, key for mid-term heat tolerance (Table 3).

At 35 DHS, considered to be long-term stress acclimation, PC1 (36.25% variance) is strongly associated with tuber traits (NT, FTW, DMC), leaf conductance, transpiration, and photosynthesis, indicating that long-term heat tolerance is driven by sustained reproductive output and gas exchange. Selection could be made for genotypes with high PC1 scores excel in tuber production and thermoregulation, critical for yield stability. PC2 (19.16%) is linked to transpiration, shoot biomass, and negatively to pigments, suggesting that some genotypes prioritize cooling and growth over pigment maintenance. PC3 (13.97%) correlates with photosynthesis and PSII efficiency, reflecting photosynthetic recovery. PC4 (8.93%) is driven by Fv/Fm and dry shoot weight, indicating photosystem and biomass stability. The negative loading of pigments on PC1 suggests a trade-off, where high-yielding genotypes may sacrifice pigment content. For breeding, selecting genotypes with high PC1 and PC3 scores would target sustained tuber yield and photosynthetic efficiency under prolonged HS (Table 3).

The PCA results highlight distinct physiological strategies for heat tolerance across timepoints, with implications for breeding heat-resilient wild potatoes. At 1 DHS, breeding should focus on genotypes with strong photosynthetic and gas exchange traits to ensure early stress tolerance, as these traits (high PC1, PC2) correlate with initial survival. At 15 DHS, selecting genotypes with balanced agronomic and photosynthetic traits (high PC1, PC2) ensures mid-term resilience, particularly for tuber yield. By 35 DHS, prioritizing genotypes with high tuber production, gas exchange, and photosynthetic recovery (high PC1, PC3) is critical for long-term yield stability under heat. The trade-offs between vegetative growth and tuber yield suggest that breeding programs should target genotypes that balance these traits, potentially using *S. chacoense* for its stronger tuber resilience and *S. commersonii* for photosynthetic stability (Table 3).

### 2.6. Ranking of Wild Potato Genotypes Based on Their Heat Tolerance

Since a single physiological index could not serve as a reliable indicator of heat tolerance in crops, heat comprehensive evaluation value (HCEV) and comprehensive principal component values (F) based on multiple indexes were used for evaluating heat tolerance (Table 4), providing a robust framework for ranking wild potato genotypes (*S. commersonii* and *S. chacoense*) based on their physiological and agronomic responses to HS. At 1 DHS, genotypes such as BGB106, BGB048, BGB100, and BGB108 rank the highest (HCEV: 0.73–0.62), reflecting strong early tolerance driven by sustained Pn, gas exchange (Gs, E), and pigment stability, as these traits dominate the PCA’s first components. BGB001 and BGB055 rank the lowest (HCEV: 0.33–0.29), indicating initial sensitivity, likely due to reduced photosynthetic efficiency and tuber initiation. By 15 DHS, BGB110, BGB108, and BGB011 lead (HCEV: 0.70–0.63), showcasing mid-term resilience through balanced tuber production, shoot biomass retention, and recovery in Pn and gas exchange, while BGB001 and BGB453 lag (HCEV: 0.20–0.27) due to persistent reductions in these traits. At 35 DHS, BGB108, BGB100, and BGB055 top the rankings (HCEV: 0.74–0.56), excelling in sustained tuber yield, gas exchange, and photosynthetic recovery, critical for long-term heat tolerance, whereas BGB077 and BGB001 rank poorly (HCEV: 0.18–0.26) due to weak tuber production and limited physiological acclimation. The F values align closely with the HCEV rankings, reinforcing that genotypes with high positive F scores (e.g., BGB108, BGB100, F_35: 0.52–0.29) exhibit robust multivariate trait performances. For breeding, selecting top-ranked genotypes like BGB108, BGB100, BGB106, and BGB110, which consistently perform well across stages, ensures the introgression of traits like photosynthetic stability, effective thermoregulation, and tuber yield resilience into cultivated potatoes. Conversely, low-ranking genotypes (e.g., BGB001, BGB077, BGB453) highlight vulnerabilities, particularly in reproductive output, guiding breeders to avoid these for heat-tolerance programs. This ranking system, integrating PCA-derived weights, offers a precise tool for identifying heat-tolerant wild potato germplasm to enhance crop resilience under rising temperatures.

### 2.7. Clustering of Wild Potato Accession Based on the Similarity Observed by the Comprehensive Values

**Figure 6:**
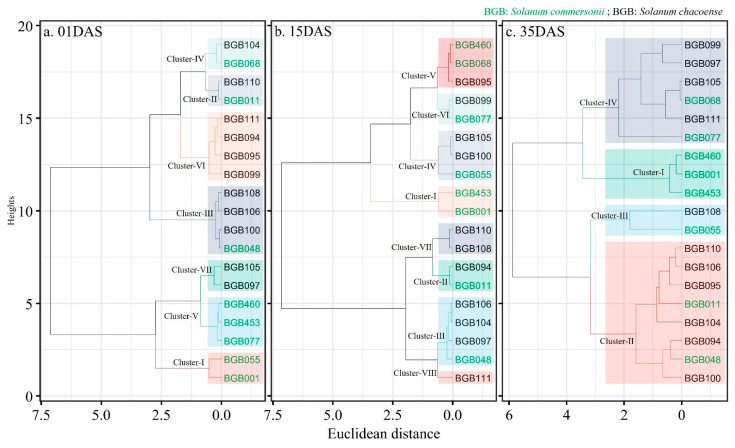
Cluster analysis for the heat tolerance of wild potato accessions based on HCEV and F value.

Cluster analysis results of 19 wild potato accessions based on the HCEV and F value were divided into different groups. Figure 6 presents a hierarchical clustering dendrogram of wild potato genotypes (*S. commersonii* and *S. chacoense*) based on their heat tolerance coefficient (HTC) values at 1, 15, and 35 days of heat HS (DHS), visualized across three panels (a, b, c). The x-axis represents Euclidean distance, indicating dissimilarity between genotypes, while the y-axis shows height, reflecting the degree of clustering. Different colors and cluster labels (I–VII) distinguish genotype groups based on their heat tolerance patterns.

At 01 DHS (Figure 4A), seven clusters emerge. Cluster-I (e.g., BGB055, BGB001) shows high dissimilarity, suggesting low early heat tolerance. Cluster-II (e.g., BGB011, BGB110) and Cluster-IV (e.g., BGB104, BGB068) indicate moderate tolerance, while Cluster-VII (e.g., BGB106, BGB100) forms a distinct group with potential early resilience, aligning with high D/F rankings.

At 15 DHS (Figure 4B), the structure shifts to seven clusters. Cluster-V (e.g., BGB460, BGB068) and Cluster-VI (e.g., BGB105, BGB100) show increased dissimilarity, reflecting diverse mid-term responses. Cluster-II (e.g., BGB011, BGB097) and Cluster-VII (e.g., BGB111) suggest improved tolerance, consistent with mid-term recovery in photosynthesis and tuber traits.

At 35 DHS (Figure 4C), five clusters are identified. Cluster-I (e.g., BGB001, BGB453) indicates low persistent tolerance, while Cluster-IV (e.g., BGB108, BGB055) and Cluster-II (e.g., BGB094, BGB048) highlight genotypes with sustained tolerance, aligning with strong gas exchange and tuber yield. Cluster-III (e.g., BGB100, BGB106) shows a tight grouping, suggesting robust long-term resilience. The evolving cluster patterns reflect dynamic heat tolerance, with *S. chacoense* (e.g., BGB108, BGB100) showing consistent resilience, especially at 35 DHS, and *S. commersonii* (e.g., BGB011, BGB048) improving over time. For breeding, the results point to prioritizing genotypes in Clusters-IV and II for long-term heat tolerance, integrating their traits into cultivated varieties to enhance climate resilience.

## 3. Discussion

By employing multivariate analysis and best linear unbiased prediction (BLUP) models, our study effectively identifies genotypes with superior heat tolerance traits, highlighting the unique contributions of physiological and morphological traits in potato breeding programs. The integration of these results underscores the importance of wild germplasm in addressing the escalating threat of HS to global potato production, particularly in the context of climate change. Morphological traits, such as fresh shoot weight (FSW), tuber number (NT), and tuber weight (TW), showed significant genotypic variation, with *S. chacoense* demonstrating greater resilience than *S. commersonii*, particularly in tuber traits, as shown in our previous studies of wild potato germplasms [27]. This is consistent with the adaptability of *S. chacoense* to arid environments, attributing its thermotolerance to efficient water use and robust root architecture [9]. Studies on other tuber crops, such as cassava, have similarly identified root and shoot biomass stability as critical for yield maintenance under HS [28]. The moderate-to-high heritability (60–90%) of tuber traits in our study aligns with our previous study [29], which concluded that wild *Solanum* species possess polygenic traits for tuberization with high heritability (60–90%) that can be introgressed into cultivated varieties. However, the significant reduction in NT and FTW under HS as compared with CT, although no significant G × E was present, mirrors findings in cultivated potatoes, as well as in other studies of wild potato accession [27,29], where HS reduces tuber initiation by 30–44% [1]. This underscores the challenge of overcoming linkage drag [11], when transferring wild traits, necessitating advanced genomic tools like those used in this study.

The significant genotypic variation in heat tolerance observed in this study corroborates findings from other investigations into wild potato relatives. For instance, *S. chacoense* exhibited superior heat tolerance compared with *S. commersonii*, particularly in maintaining photosynthesis and tuber production under prolonged HS (Figure 4; Table S3), consistent with Bashir et al. [27]. Photosynthetic efficiency and chlorophyll stability are robust indicators of heat tolerance across crops like wheat and maize, where genotypes maintaining higher Pn under elevated temperatures sustain greater assimilate supply to yield components [13]. Similarly, in wild potato relatives, genotypes like BGB108 and BGB100 exhibited enhanced Pn and Gs by 35 (DHS), with heat tolerance coefficients (HTCs) exceeding 1, indicating adaptive responses akin to those observed in heat-tolerant wheat varieties [15]. Wild-derived potato lines, particularly from *S. chacoense*, maintained tuber sets under HS due to sustained photosynthetic capacity [10]. The high heritability (89–98%) of PSII efficiency and Fv/Fm ratio in this study further supports that stable photosystem function is a hallmark of thermotolerance in crops like tomato, a close relative of potato [14]. These parallels suggest that physiological traits prioritized in this study are broadly applicable across species, reinforcing their utility in breeding programs.

Comparatively, studies on other crops provide additional context for the physiological mechanisms observed. Wild relatives of other crops, such as tomato (*Solanum pimpinellifolium*), exhibit superior thermotolerance due to enhanced stomatal regulation and pigment stability [12]. The increased chlorophyll and carotenoid content under HS, particularly at 35 DHS, throughout the whole germplasm used, mirrors findings in chickpea, where pigment accumulation supports photoprotection under high temperatures [30]. However, the trade-off between vegetative growth and tuber yield, as seen in genotypes like BGB055, reflects a common stress response in tuber crops, where resource allocation prioritizes survival over reproduction [17]. This trade-off necessitates careful selection to balance vegetative and reproductive resilience [16]. In wheat, photosynthetic efficiency and chlorophyll stability were identified as key indicators of heat tolerance [13], mirroring the high heritability (89–98%) and genetic advance observed for PSII efficiency (YII) and Fv/Fm in our study (Figure 1). Similarly, in rice, genotypes with enhanced transpiration and leaf conductance under HS maintained yield stability [31], akin to the high heat tolerance coefficient (HTC > 1) for transpiration (E) and leaf conductance (Gs) for genotypes like BGB048 and BGB094 (Figure 4 and Figure 5, Table 2). These parallels suggest that traits conferring heat tolerance, such as efficient gas exchange and photoprotection, are conserved across species, reinforcing the utility of wild potato relatives in breeding programs. However, unlike cereals, potatoes face unique challenges due to their tuber-based reproductive strategy, which is highly sensitive to heat-induced assimilate translocation impairments [1]. Our findings that tuber traits (NT, FTW) exhibited high heritability (60–90%) but significant reductions under HS (up to 44% for FTW), underscoring the need to prioritize genotypes like BGB108 and BGB110, which maintain tuber production (Table S3).

The temporal dynamics of HS responses in this study, particularly the acclimation observed by 35 DHS, reflect adaptive physiological mechanisms that have been documented in other stress-tolerant crops. For instance, the significant increase in photosynthesis (HTC = 1.32), leaf conductance (HTC = 2.01), and transpiration (HTC = 2.77) under prolonged HS aligns with findings in sorghum, where late-stage acclimation to HS was linked to enhanced stomatal regulation and photosynthetic recovery [32]. The stability of PSII efficiency (YII) and Fv/Fm in genotypes like BGB100 and BGB108 mirrors results in maize, where heat-tolerant lines maintained photochemical efficiency under stress [33]. These conserved mechanisms suggest that wild potato relatives, particularly *S. chacoense*, could serve as a model for understanding heat tolerance across tuber and non-tuber crops. Similar responses for *S. chacoense* were also observed by [27], where the accessions were maintaining or conserving their energy toward final yield. However, the trade-off between vegetative growth (FSW, DSW) and tuber production observed in the PCA results, where high-yielding genotypes often sacrificed shoot biomass, echoes findings in tomato, where HS redirected assimilates from vegetative to reproductive sinks [34]. This trade-off necessitates careful selection by using multivariate analysis, mixed models, and genetic parameters in breeding programs to balance vegetative vigor and tuber yield.

The use of multivariate analysis, particularly BLUP models, principal component analysis (PCA) and cluster analysis, enhances the understanding of trait interactions and their implications for breeding. The PCA results (Table 3) identified photosynthesis, gas exchange, and tuber traits as dominant contributors to variance at different stress, which collaborate with other multivariate approaches to dissect complex trait interactions in crops such as barley [20]. The identification of genotypes like BGB108 and BGB100 as highly tolerant, with high HCEV and F values at 35 DHS (Table 4), reflects their ability to balance photosynthetic efficiency and tuber yield, a strategy also observed in heat-tolerant rice varieties [31]. The clustering of genotypes into distinct groups based on heat tolerance coefficients (HTCs) and comprehensive evaluation values (HCEVs) parallel studies on potato and maize, where cluster analysis segregated heat-tolerant accessions based on physiological and yield traits [35,36]. These methodologies provide a robust framework for selecting elite genotypes, addressing genotype-by-environment (G × E) interactions as critical in HS studies [21].

The application of BLUP models to predict genotypic values further strengthens the study’s breeding implications, as it accounts for environmental noise and G × E interactions, consistent with de Resende et al. [25]. The high heritability and genetic advance observed for traits like Gs and E (47–93% and up to 120% at 15 DHS) indicate substantial genetic variability for selection, similar to findings in maize where BLUP enhanced selection accuracy for heat tolerance [33], supporting their integration into marker-assisted selection (MAS) [12]. The integration of BLUP with genomic selection [26], could accelerate trait introgression in potatoes, particularly for polygenic traits like heat tolerance. This approach is particularly relevant given the significant G × E interactions observed for YII, NPQ, and Fv/Fm, which highlight the need for stable performers across diverse conditions [37] because it allows early, accurate selection of the heat-tolerant accessions, which with consistent performance, could speed up the breeding cycle.

The importance of wild potato relatives in breeding programs extends beyond heat tolerance to broader abiotic stress resilience. For instance, the ability of *S. chacoense* to maintain pigment content (HTC > 1 for Chl-A, Chl-B, and carotenoids) under HS parallels drought-tolerant traits in wild tomato relatives (*Solanum chilense*), which exhibit enhanced carotenoid accumulation for photoprotection [18]. Similarly, the high heritability of tuber traits (Table 1) in this study aligns with findings in wild yam (*Dioscorea* spp.), where tuber quality traits showed strong genetic control under stress [38,39]. These cross-crop comparisons highlight the potential of wild relatives to introduce novel alleles for stress tolerance, addressing genetic narrowing in cultivated potatoes [6]. However, challenges such as crossability barriers and linkage drag [11], remain significant hurdles. The success of genotypes like BGB108 and BGB100 in this study suggests that targeted introgression, coupled with genomic tools [26], can mitigate these issues by precisely transferring heat-tolerant alleles.

The clustering of genotypes into highly tolerant, moderately tolerant, sensitive with late recovery, and highly sensitive groups based on HCEV and F values provides a practical framework for breeders. Highly tolerant genotypes like BGB108 and BGB100, which excel in photosynthesis, gas exchange, and tuber resilience, are prime candidates for introgression into cultivated varieties. Their performance aligns with findings in wild barley (*Hordeum spontaneum*), where top-performing genotypes under HS were prioritized for breeding due to their balanced physiological and yield traits [40]. Conversely, sensitive genotypes like BGB001 and BGB453 highlight vulnerabilities that breeders should avoid, particularly their poor tuber production, which mirrors heat-sensitive potato cultivars [2]. The integration of physiological traits (e.g., Gs, YII) with agronomic outcomes (NT, FTW) in the HCEV and F rankings underscores the need for a holistic approach to breeding, as single-trait selection may overlook critical interactions [16,27].

In the context of potato breeding, the results emphasize the critical role of wild relatives in addressing climate change challenges. The significant reductions in tuber yield (up to 44% for FTW) under HS, coupled with the CIP’s warning of potential 25% global yield losses [5], highlight the urgency of incorporating heat-tolerant traits. The physiological stability of *S. chacoense* genotypes, particularly their enhanced transpiration and PSII efficiency, offers a pathway to develop cultivars that maintain yield under rising temperatures. These traits could be particularly impactful in tropical and subtropical regions, where HS exacerbates yield losses [3]. Furthermore, the high genetic variability (GCV up to 44% for Pn) and heritability observed in this study suggest that wild potato relatives can contribute to long-term breeding goals, for enhancing food security in developing nations [4].

## 4. Materials and Methods

### 4.1. Plant Material and HS Application

The experiment was carried out in growth chambers of the phenotyping platform from Embrapa Clima Temperado, in Pelotas, Rio Grande do Sul (RS), Brazil (32°45′ S and 52°30′ W), in a randomized complete block, factorial arrangement with 2 biological replicates. A total of 19 wild species accessions belonging to *Solanum commersonii* Dunal and *Solanum chacoense* Bitter, introduced or collected from different origins and conserved at Embrapa Potato Genebank (Table S1), were evaluated. Ten uniform tubers from the randomly selected accessions were taken out of cold chambers and placed on phenolic foam for 20 days. After acclimatization, one tuber was transplanted into a 6 L plastic pot filled with approximately 5 kg of organo-mineral substrate composed of peat and calcite limestone, added with N (0.04%), P_2_O_5_ (0.04%), and K_2_O (0.05%) under both. The plants were kept in a greenhouse for the following 15 days until they reached the emergence stage. After that, three uniform plants were transferred to growth chambers, ensuring complete randomization. Hereafter, plants were exposed to two temperature gradients: the control treatment (CT), with a daily amplitude of 14 to 27 °C, which is the temperature range for the optimum growth for potato crops, and the HS treatment, with a daily amplitude of 24 to 34 °C. The photoperiod was 12 h (7:00 to 19:00 h) with a light intensity of 400 μmol m^−2^ s^−1^ and relative humidity was maintained between 50 and 60% throughout the experiment (Table 5). The accession remained under these conditions for 60 days, until harvest time.

### 4.2. Measurement of Photosynthetic and Chlorophyll Fluorescence Parameters

Photosynthesis parameters were determined using the Li-6400XT portable photosynthetic meter (6400-02B; Li-Cor, Lincoln, NE, USA), and chlorophyll fluorescence was measured with an IMAGING-PAM M-Series 500 fluorometer (Walz Heinz GmbH, Effeltrich, Germany) after 1, 15, and 35 days of high temperature treatment. Parameter observations for photosynthesis (Pn) µmol CO_2_ m^−2^ s^−1^, leaf conductance (Gs) mol H_2_O m^−2^ s^−1^, and transpiration (E) mmol H_2_O m^−2^ s^−1^ were taken from the upper leaves, and from each biological replicate, two readings were taken. Chlorophyll fluorescence traits such as PSII efficiency (YII), heat quenching (NPQ), and PSII quantum efficiency (Fv/Fm) were measured by taking leaf samples from the upper parts of the wild potato plants. Before evaluations, in both growth chambers (CT and HS), potato plants were kept at constant temperatures of 24 °C and 34 °C, respectively and lights were turned off for dark adaptation, and the determination time was optimal from 8:30 to 11:30 a.m.

### 4.3. Measurement of Pigment Traits

The fourth fully unfolded leaf of each plant from the top down was sampled. The chlorophyll content was calculated according to the formula of Wellburn [41].

(1) $$\mathrm{Chlorophyll}\text{-}A\;\left(\mathrm{Chl}\text{-}A\right)=\left(12.19\times A_{665}\right)-\left(3.45\times A_{649}\right)$$

(2) $$\mathrm{Chlorophyll}\text{-}B\;\left(\mathrm{Chl}\text{-}B\right)=\left(21.99\times A_{649}\right)-(5.32\times A_{665})$$

(3) $$\mathrm{Carotenoid}\;\mathrm{content}\;\left(\mathrm{Cart}\right)=\left[\left(10^{3}\times A_{480}\right)-\left(2.14\times {\mathrm{Chl}}_{A}\right)-\left(70.16\times {\mathrm{Chl}}_{B}\right)\right]/220$$

### 4.4. Measurement of Tuber Traits

At the end of the potato plant life cycle, tubers were collected and counted per plant (NT) and the yield of fresh tubers (TW) was measured using an analytical balance (precision 0.0001 g). The dry matter contents (DMCs) of tubers were determined by oven drying at 70 °C for three days, and the final average values were calculated by oven dry weight ÷ initial fresh weight × 100.

### 4.5. Measurement of Biomass

The aerial parts of potato plants were sampled and cleaned separately after harvest. The fresh weight of shoot (FSW) was measured using an analytical balance (precision 0.0001 g). Dry weight (DSW) was measured after drying the arial parts in an oven at 80 °C until a constant weight was achieved.

### 4.6. Genotypic Values Prediction

The best linear unbiased predicted (BLUP) model was used to predict the true genotypic values for 1, 15 and 35 DHS, separately. The original observed values of the measured physiological and morphological traits were submitted to SELEGEN Software V1.0 [42] using the procedure suggested by Resende and Duarte [25]

The following mixed model ‘Y = Xr + Zg + Wi + e’ was used to separate the true genetic effects from environmental noise for predicting true genotypic values.

‘Y’ is the data vector, ‘r’ is the vector of repetition effects (assumed to be fixed) added to the overall mean, ‘g’ is the vector of genotypic effects (random), ‘e’ is the vector of residuals (random), and ‘i’ is the vector of the effects of the genotype × treatment (random).

### 4.7. Heat Tolerance Analysis

The heat tolerance coefficient (HTC) was the ratio of HS value to CT value [43]. The calculation formula of the membership function value µ(Xij) and the heat comprehensive evaluation value (HCEV) are shown as follows [36]:

(4) $$\mathrm{CHTC}=\frac{1}{\;n}\sum_{i=1}^{n}\mathrm{HTC}$$

(5) $$µ(X_{\mathrm{ij}})=(X_{\mathrm{ij}}-X_{\min})/{(X}_{\max}-X_{\min})$$

(6) $$W_{j}=V_{j}/\sum_{j=1}^{n}V_{j}$$

(7) $$F=\sum_{i=1}^{n}\left[X_{\mathrm{ij}}\times W_{j}\right]$$

(8) $$\mathrm{HCEV}=\sum_{i=1}^{n}\left[µ(X_{\mathrm{ij}})\times W_{j}\right]$$

where Xij was the value of ith accession in the jth indicator; Xmin, Xmax, and Vj indicated the minimum value, maximum value, and standard deviation coefficient for the jth indicator of all the accessions, respectively; and Wj represented the yield of the jth indicator in all indicators. HTC and CHTC indicated the heat tolerance coefficient and comprehensive heat tolerance coefficient.

### 4.8. Data Analysis

Single and double factor analysis of variance (ANOVA) and stepwise regression analysis were performed using R program [44], and the significant difference between treatments was identified based on the LSD test at *p* ≤ 0.05. The FactoMineR package [45] was used to perform the PCA and cluster analysis. The correlations among different measures were estimated by calculating Pearson correlation coefficients using the Corrplot package [46]. Meanwhile, the ggplot2 package [47] was used to draw the figures. Genotypic values were estimated by using SELEGEN software [42]. Genetic parameters such as broad sense heritability (h2bs), genotypic coefficient (GCV%), phenotypic coefficient (PCV%), and genetic gain (GG%) were calculated manually in Excel.

## 5. Conclusions

Our study on wild potato accessions (*S. commersonii* and *S. chacoense*) under CT and HS conditions reveal significant genetic and environmental influences on physiological and agronomic traits, with implications for breeding heat-tolerant potato varieties. We concluded that HS initially disrupts traits like photosynthesis (Pn), leaf conductance (Gs), and transpiration (E), particularly at 1 and 15 DHS, but many genotypes exhibit acclimation by 35 DHS, showing enhanced photosynthetic rates, gas exchange, and pigment content (chlorophyll-A, chlorophyll-B, carotenoids). Traits such as PSII efficiency (YII) and Fv/Fm demonstrate high heritability (89–99%) and stability, indicating robust photosystem resilience, while transpiration and leaf conductance show high genetic advance (GA%) and variability (GCV/PCV), suggesting strong potential for selection in breeding programs.

*S. chacoense* generally exhibits greater heat tolerance than *S. commersonii*, with better maintenance of tuber yield and gas exchange under prolonged stress. Principal component analysis (PCA) highlights that early tolerance (1 DHS) is driven by photosynthetic stability and gas exchange, mid-term tolerance (15 DHS) by biomass retention, and long-term tolerance (35 DHS) by sustained tuber yield and thermoregulation. Genotypes like BGB108, BGB100, BGB106, and BGB110 consistently rank high in heat comprehensive evaluation values (HCEVs) and comprehensive principal component (F) scores, excelling in photosynthetic efficiency, gas exchange, and tuber resilience, making them prime candidates for breeding.

Cluster analysis further groups genotypes into highly tolerant (e.g., BGB108, BGB100), moderately tolerant, sensitive with late recovery, and highly sensitive categories, with *S. chacoense* dominating the tolerant groups. Stepwise regression identifies dry shoot weight (DSW), Fv/Fm, and leaf conductance as critical traits for heat tolerance across stages. However, the trade-offs between vegetative growth and tuber yield suggest that breeding programs should prioritize genotypes balancing these traits, particularly from *S. chacoense* for tuber resilience and *S. commersonii* for photosynthetic stability.

Moreover, the study underscores genetic diversity in wild potato germplasm and the potential to enhance cultivated potato resilience to rising temperatures by selecting genotypes with high HCEV/F values and targeting early and sustained stress responses, especially in gas exchange, photosynthetic efficiency, and tuber production.

## Figures and Tables

**Table 1:** Statistical analysis of agronomic indexes in potato wild relatives (*Solanum chacoense* and *S. commersonii*, Solanaceae) from Embrapa Potato Genebank under heat stress. $\bar{x}$ ± sd, mean of 19 genotypes under CT, control; HS, heat stress; sd, ± standard deviation; HTC, heat-tolerant coefficient; G, genotype; T, treatment; G × T, interaction effect; * *p* ≤ 0.05; ** *p* ≤ 0.01; *** *p* ≤ 0.001; ns = non-significant *p* > 0.05 for fresh shoot weight (FSW) g, dry shoot weight (DSW) g, number of tubers (NTs), tuber weight (TW) g, dry matter (DM) %.

Trait	Temp.	BLUP Variation	$\bar{x}$ ± sd	$\bar{x}$ HTC	G	T	G × T	GCV %	PCV %	H^2^	GA%
FSW (g)	CT	144.9–284.6	211.6 ± 36.8	0.83	***	**	ns	316.0	21.7	44.9	30.0
	HS	109.3–247.2	176.3 ± 36.4					273.0	22.0	41.7	29.2
DSW (g)	CT	26.1–37.8	32.1 ± 3.1	0.97	***	ns	ns	62.0	10.8	26.7	11.5
	HS	25.0–36.8	31.0 ± 3.1					44.8	13.2	47.9	18.8
NT	CT	3.1–40.2	17.8 ± 11.1	0.56	***	**	ns	23.0	68.1	59.8	108.5
	HS	0.1–33.4	12.5 ± 10.2					13.4	92.9	85.8	177.2
TW (g)	CT	15.4–158.8	69.9 ± 48.1	0.29	***	***	ns	82.5	81.5	71.8	142.2
	HS	7.6–122.2	39.4 ± 42.7					41.5	100.0	89.9	195.3
DM (%)	CT	3.2–30.4	20.9 ± 8.5	0.76	***	*	ns	22.4	47.1	86.9	90.4
	HS	0.7–27.1	17.9 ± 8.5					41.5	100.0	89.9	195.3

**Table 2:** Statistical analysis of physiological indexes in potato wild relatives (*Solanum chacoense* and *S. commersonii*, Solanaceae) from the Embrapa Potato Genebank under heat stress. Mean ± sd, mean of 19 genotypes under CT, control; HS, heat stress; sd, ± standard deviation; CT vs. HS comparison by *t*-test; ** *p* ≤ 0.01; *** *p* ≤ 0.001; ns = non-significant *p* > 0.05; HTC, heat-tolerant coefficient of photosynthesis (Pn) µmol CO_2_ m^−2^ s^−1^, leaf conductance (Gs) mol H_2_O m^−2^ s^−1^, transpiration (E) mmol H_2_O m^−2^ s^−1^, PSII efficiency (YII), heat quenching (NPQ), PSII quantum efficiency (Fv/Fm), chlorophyll-A (Chl-A) mg g^−1^ FW, chlorophyll-B (Chl-B) mg g^−1^ FW, and carotenoid (Cart) mg g^−1^ FW after 1 DHS, 15 DHS, and 35 days of heat stress (DHS).

Trait	Temp.	1 DHS				15 DHS				35 DHS			
		BLUP Variation	Mean ± SD	*t*-Test	HTC Mean	BLUP Variation	Mean ± SD	*t*-Test	HTC Mean	BLUP Variation	Mean ± SD	*t*-Test	HTC Mean
Pn	CT	5.24–15.91	11.16 ± 2.70	ns	1.02	1.94–13.12	8.49 ± 3.50	**	0.77	5.56–10.00	7.84 ± 1.10	***	1.32
	HS	8.29–13.18	10.75 ± 1.40			1.23–12.40	6.29 ± 3.60			7.82–13.23	10.24 ± 1.3		
Gs	CT	0.02–0.29	0.12 ± 0.10	ns	1.15	0.02–0.39	0.16 ± 0.10	**	0.60	0.06–0.27	0.14 ± 0.10	***	2.01
	HS	0.03–0.33	0.13 ± 0.10			0.01–0.28	0.08 ± 0.10			0.15–0.47	0.27 ± 0.10		
E	CT	0.45–3.40	1.78 ± 0.80	ns	1.09	0.30–3.92	2.00 ± 1.20	ns	1.06	0.87–2.64	1.8 ± 0.50	***	2.77
	HS	0.46–3.62	1.88 ± 0.90			0.29–5.45	1.88 ± 1.60			3.44–6.42	4.77 ± 0.70		
YII	CT	0.03–0.19	0.11 ± 0.04	ns	1.02	0.03–0.21	0.12 ± 0.05	ns	1.03	0.02–0.19	0.12 ± 0.10	***	2.42
	HS	0.05–0.22	0.09 ± 0.03			0.06–0.19	0.11 ± 0.04			0.08–0.28	0.18 ± 0.10		
NPQ	CT	0.45–0.85	0.67 ± 0.10	ns	1.01	0.42–0.90	0.65 ± 0.10	ns	1.04	0.50–0.91	0.69 ± 0.10	***	0.86
	HS	0.40–0.85	0.67 ± 0.10			0.43–0.82	0.67 ± 0.10			0.36–0.74	0.59 ± 0.10		
Fv/Fm	CT	0.50–0.79	0.75 ± 0.10	**	0.95	0.59–0.78	0.75 ± 0.10	ns	1.02	0.71–0.80	0.76 ± 0.02	ns	0.99
	HS	0.59–0.78	0.70 ± 0.10			0.73–0.78	0.76 ± 0.01			0.69–0.79	0.75 ± 0.03		
Chl-A	CT	1.47–1.60	1.52 ± 0.03	***	0.90	1.62–2.19	1.89 ± 0.20	***	1.09	1.68–2.18	1.92 ± 0.20	***	1.17
	HS	1.33–1.44	1.37 ± 0.02			1.79–2.35	2.05 ± 0.20			1.99–2.50	2.24 ± 0.20		
Chl-B	CT	0.61–0.73	0.66 ± 0.03	***	0.89	0.74–0.88	0.82 ± 0.04	***	1.02	0.59–0.84	0.73 ± 0.10	***	1.23
	HS	0.56–0.67	0.59 ± 0.02			0.76–0.90	0.83 ± 0.04			0.75–1.00	0.90 ± 0.10		
Cart	CT	0.25–0.43	0.31 ± 0.04	ns	0.98	0.33–0.43	0.38 ± 0.03	***	1.11	0.36–0.40	0.38 ± 0.01	***	1.09
	HS	0.25–0.37	0.30 ± 0.03			0.37–0.47	0.42 ± 0.03			0.40–0.44	0.42 ± 0.01		

**Table 3:** Loading matrix and the variance contribution rate of the principal component under heat stress (HS) as evaluated in wild accessions from Embrapa Potato Genebank. PC, principal component; CR, contribution rate; CCR, cumulative contribution rate at 1, 15, 35 days of heat stress (DHS).

Index	1 DHS						15 DHS				
	PC1	PC2	PC3	PC4	PC5	Total Weight	PC1	PC2	PC3	PC4	Total Weight
Photosynthesis	0.61	0.40	0.46	0.03	−0.30	0.19	−0.34	0.82	0.23	−0.09	0.09
Leaf conductance	0.22	0.80	0.33	−0.34	0.14	0.17	−0.29	0.81	0.11	−0.37	0.05
Transpiration	0.23	0.80	0.21	−0.37	0.31	0.17	−0.32	0.88	0.12	−0.28	0.07
PSII efficiency	0.30	0.12	0.49	0.66	−0.31	0.16	0.09	0.56	−0.31	0.47	0.11
Heat quenching	−0.10	−0.59	−0.07	0.03	0.65	−0.04	0.36	−0.04	0.09	−0.40	0.03
PSII quantum efficiency	0.14	−0.20	0.68	0.46	0.24	0.14	0.25	0.31	−0.11	0.69	0.16
Chlorophyll-A	0.68	−0.53	0.40	−0.22	−0.02	0.07	−0.04	0.02	0.93	0.32	0.17
Chlorophyll-B	0.62	−0.50	0.41	−0.40	0.01	0.05	0.19	0.07	0.77	−0.03	0.16
Carotenoid	0.77	−0.46	0.19	−0.26	−0.02	0.07	0.13	0.04	0.87	0.32	0.20
Fresh shoot weight	−0.67	0.21	0.47	0.04	0.12	−0.02	−0.81	0.10	−0.17	−0.02	−0.15
Dry shoot weight	−0.53	0.06	0.49	0.11	0.50	0.03	−0.62	0.20	−0.33	0.56	−0.06
Number of tubers	0.76	0.20	−0.33	0.33	0.26	0.18	0.80	0.44	−0.29	0.06	0.17
Fresh tuber weight	0.82	0.13	−0.34	0.29	0.26	0.17	0.87	0.34	−0.19	0.03	0.18
Dry matter	0.77	0.35	−0.28	0.09	0.22	0.18	0.85	0.26	−0.01	−0.09	0.18
Eigenvalue	4.61	2.82	2.18	1.38	1.23		3.74	2.95	2.67	1.60	
CR (%)	32.95	20.13	15.55	9.89	8.77		26.69	21.11	19.04	11.42	
CRR (%)	32.95	53.08	68.63	78.52	87.29		26.69	47.80	66.84	78.26	
Weight	0.38	0.23	0.18	0.11	0.10		0.34	0.27	0.24	0.15	
	**35 DHS**										
Photosynthesis	0.53	0.25	0.69	0.00	−0.31	0.19					
Leaf conductance	0.66	0.50	0.47	−0.11	−0.05	0.23					
Transpiration	0.51	0.64	0.48	−0.07	0.16	0.25					
PSII efficiency	−0.23	0.05	0.48	−0.11	0.68	0.07					
Heat quenching	0.45	0.30	−0.53	−0.18	0.25	0.07					
PSII quantum efficiency	0.63	0.05	−0.13	0.65	0.12	0.18					
Chlorophyll-A	−0.75	−0.40	0.37	0.12	0.03	−0.14					
Chlorophyll-B	−0.50	−0.52	0.41	0.02	0.39	−0.08					
Carotenoid	−0.77	−0.14	0.32	0.16	−0.35	−0.14					
Fresh shoot weight	−0.45	0.63	−0.15	0.16	0.26	0.02					
Dry shoot weight	−0.41	0.43	−0.03	0.77	0.06	0.06					
Number of tubers	0.76	−0.48	0.20	0.28	−0.01	0.13					
Fresh tuber weight	0.80	−0.49	0.13	0.15	0.01	0.11					
Dry matter	0.66	−0.59	−0.14	0.04	0.23	0.05					
Eigenvalue	5.07	2.68	1.96	1.25	1.06						
CR (%)	36.25	19.16	13.97	8.93	7.58						
CRR (%)	36.25	55.40	69.37	78.31	85.89						
Weight	0.42	0.22	0.16	0.10	0.09						

**Table 4:** Ranking of wild potato germplasm based on the HCEV and F values under heat stress.

Genotype	1 DHS				15 DHS				35 DHS			
	HCEV Value	Rank	F Value	Rank	HCEV Value	Rank	F Value	Rank	HCEV Value	Rank	F Value	Rank
BGB001	0.33	18	−1.68	18	0.20	19	−1.95	19	0.26	17	0.23	6
BGB011	0.62	7	0.49	7	0.63	3	1.00	3	0.42	11	−0.08	12
BGB048	0.72	2	1.24	2	0.59	5	0.68	5	0.53	5	0.19	8
BGB055	0.29	19	−2.08	19	0.34	17	−1.14	17	0.56	3	1.08	1
BGB068	0.65	6	0.73	6	0.43	14	−0.34	13	0.36	13	−0.29	15
BGB077	0.42	17	−0.88	16	0.47	10	−0.08	11	0.18	19	−0.83	19
BGB094	0.57	10	0.08	10	0.62	4	0.94	4	0.54	4	0.36	3
BGB095	0.55	11	−0.06	12	0.45	12	−0.29	12	0.46	10	0.22	7
BGB097	0.47	14	−0.39	13	0.57	7	0.68	6	0.41	12	−0.77	18
BGB099	0.51	12	0.01	11	0.46	11	0.02	10	0.48	9	−0.55	17
BGB100	0.72	3	1.30	1	0.37	16	−0.72	15	0.62	2	0.29	5
BGB104	0.67	5	0.81	5	0.57	8	0.55	7	0.53	6	−0.18	13
BGB105	0.50	13	−0.58	14	0.38	15	−0.72	16	0.36	14	−0.27	14
BGB106	0.73	1	1.07	4	0.58	6	0.53	8	0.51	7	0.06	11
BGB108	0.71	4	1.15	3	0.68	2	1.30	1	0.74	1	0.52	2
BGB110	0.61	8	0.38	8	0.70	1	1.30	2	0.48	8	0.09	10
BGB111	0.58	9	0.14	9	0.53	9	0.28	9	0.34	15	−0.50	16
BGB453	0.44	15	−0.92	17	0.27	18	−1.61	18	0.26	18	0.11	9
BGB460	0.44	16	−0.81	15	0.43	13	−0.42	14	0.28	16	0.31	4

HCEV, heat comprehensive evaluation value, F, comprehensive principal components value. The number in each column represents the score of principal components that were calculated based on mean (n = 3 biological replicates).

**Table 5:** Growth chambers control and heat stress environmental conditions, with controlled photoperiods, temperature, and humidity.

Chamber-1: Control			Chamber 2: Heat Stress		
Time	Temperature °C	Humidity %	Time	Temperature °C	Humidity %
00:00–04:00	19	65	23:00–01:00	27	65
04:00–06:00	15	65	01:00–04:00	26	65
06:00–09:00	14	65	04:00–06:00	25	65
09:00–10:00	16	50	06:00–09:00	24	50
10:00–11:00	19	50	09:00–11:00	27	50
11:00–12:00	23	50	11:00–12:00	30	50
12:00–14:00	25	50	12:00–14:00	31	50
14:00–18:00	27	50	14:00–18:00	34	50
18:00–21:00	26	50	18:00–21:00	31	50
21:00–00:00	23	65	21:00–23:00	28	65

## Data Availability

Data are contained within the article and Supplementary Materials.

## Supplementary Materials

The following supporting information can be downloaded at https://www.mdpi.com/article/10.3390/plants14193096/s1. Figure S1. Mean comparison of 19 wild potato genotypes between control (CT) and heat stress (HS) condition for Photosynthesis (Pn) µmol CO_2_ m^−2^ s^−1^, Leaf conductance (Gs) mol H_2_O m^−2^ s^−1^, Transpiration (E) mmol H_2_O m^−2^ s^−1^, PSII efficiency (YII), Heat quenching (NPQ), PSII quantum efficienty (Fv/Fm), Chlorophyll-A (Chl-A) mg g^−1^ FW, Chlorophyll-B (Chl-B) mg g^−1^ FW, Carotenoid (Cart) mg g^−1^ FW, Fresh shoot weight (FSW) g, Dry shoot weight (DSW) g, Number of tubers (NT), Tuber weight (TW) g, dry matter (DM) %. Figure S2. Wild potato genotypes of S. chacoense and S. commersonii from Embrapa Potato Gene-bank performance as heat tolerance coefficient under control temperature (CT) and heat stress (HS) conditions after 1, 15 and 35 DHS, for chlorophyll-A (Chl-A), chlorophyll-B (Chl-B), and carotenoid (Cart). Figure S3. *Solanum commersonii* accessions after 35 days of heat stress under control and heat stress conditions, and tuber production under both conditions. Figure S4. *Solanum chacoense* accessions after 35 days of heat stress under control and heat stress conditions, and tuber production under both conditions. Table S1. Wild potato germplasm (Solanum) from Embrapa Potato Genebank used for screening under heat stress. Table S2. Analysis of variance for physiological and agronomical traits measured after 1, 15, and 35 days in 19 different wild genotypes of potato at 2 different temperature regimes. Table S3. Heat tolerance coefficient of potato wild relatives (Solanum) calculated from BLUP values predicted under control and heat stress conditions for fresh shoot weight (FSW), dry shoot weight (DSW), number of tubers (NT), tuber weight (TW), and dry matter (DM).
