# Supplementary material for: A Methodological Approach for Evaluating the Genotypic Variation for Physiological Adaptation of Potato Wild Relatives for Heat Tolerance Breeding
Source: Plants (Basel). 2025 Oct 8;14(19):3096. doi: 10.3390/plants14193096 (PMC12526108; doi:10.3390/plants14193096)
Supplement: Supplementary file 1 [file plants-14-03096-s001.zip › plants-3823632-Supplementary.pdf]

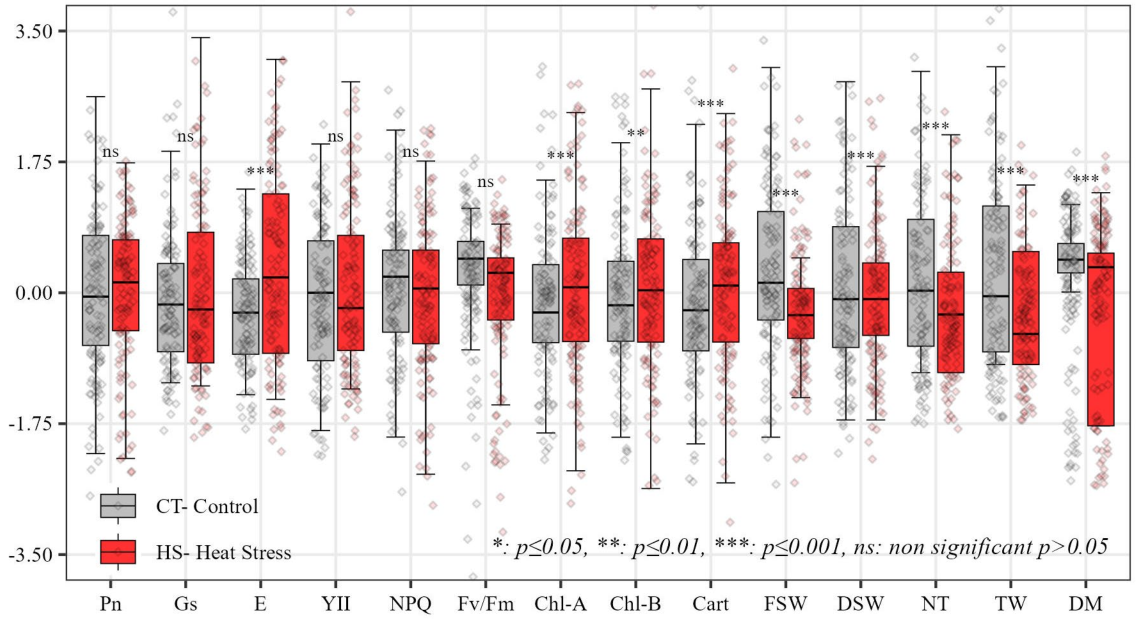

Figure S. 1: Mean comparison of 19 wild potato genotypes between control (CT) and heat stress (HS) condition for Photosynthesis (Pn) µmol CO_2 $m^{-2}s^{-1}$, Leaf conductance (Gs) mol H_2O $m^{-2}s^{-1}$, Transpiration (E) mmol H_2O $m^{-2}s^{-1}$, PSII efficiency (YII), Heat quenching (NPQ), PSII quantum efficiency (Fv/Fm), Chlorophyll-A (Chl-A) mg g^{-1} FW, Chlorophyll-B (Chl-B) mg g^{-1} FW, Carotenoid (Cart) mg g^{-1} FW, Fresh shoot weight (FSW) g, Dry shoot weight (DSW) g, Number of tubers (NT), Tuber weight (TW) g, dry matter (DM) %.

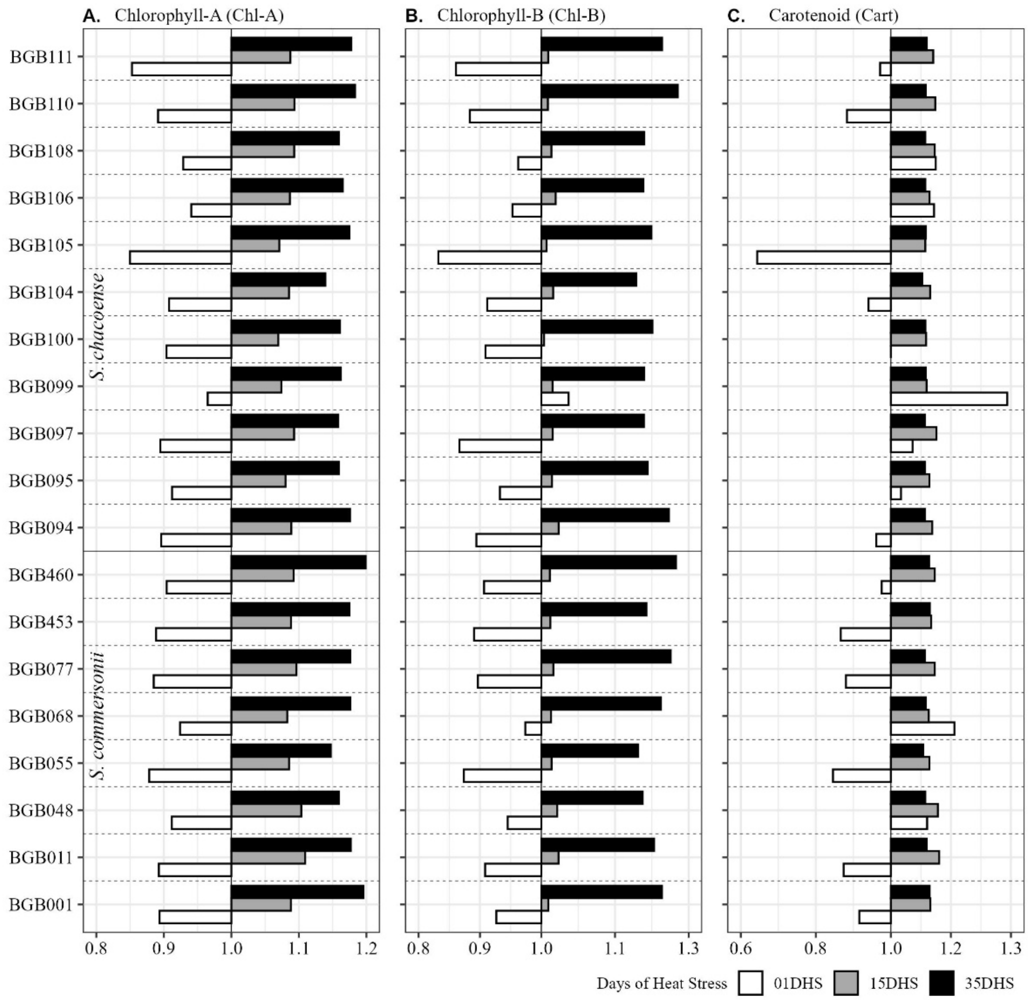

Figure S. 2: Wild potato genotypes of S. chacoense and S. commersonii from Embrapa Potato Gene-bank performance as heat tolerance coefficient under control temperature (CT) and heat stress (HS) conditions after 1, 15 and 35 DHS, for chlorophyll-A (Chl-A), chlorophyll-B (Chl-B), and carotenoid (Cart).

S. commersonii	Control	Stress	Tuber Production	*S. commersonii*	Control	Stress	Tuber Production
BGB001	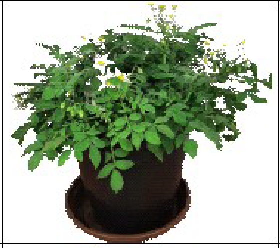	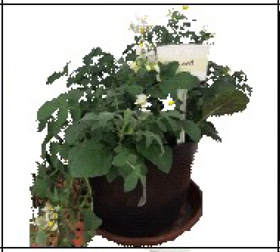	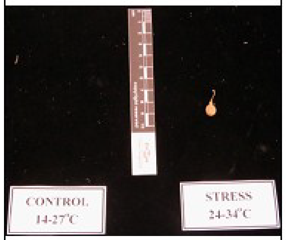	BGB068	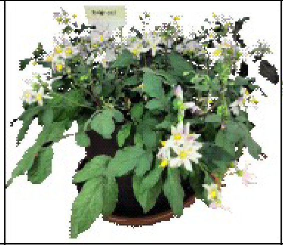	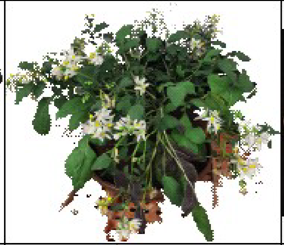	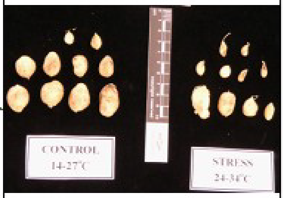
BGB011	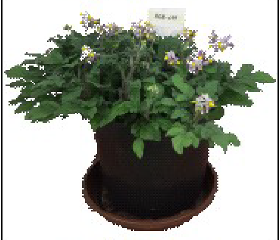	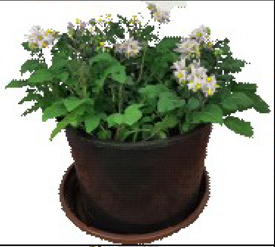	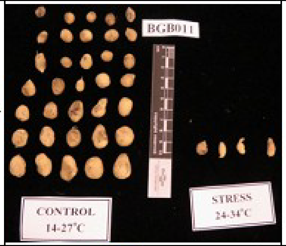	BGB077	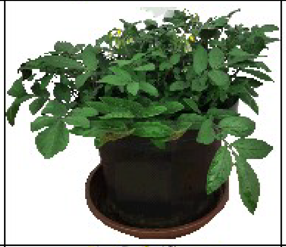	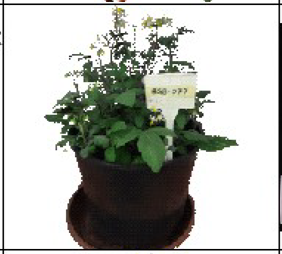	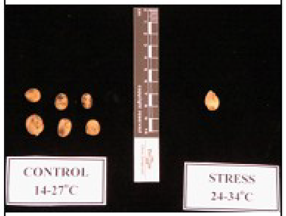
BGB048	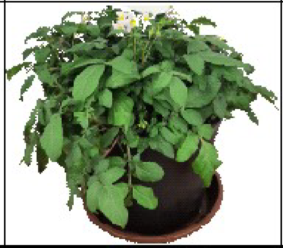	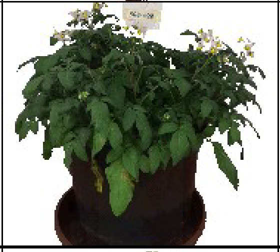	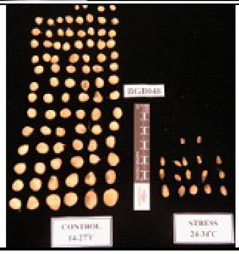	BGB453	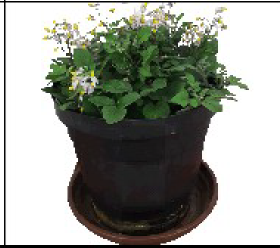	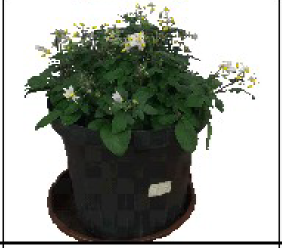	No tuber
BGB055	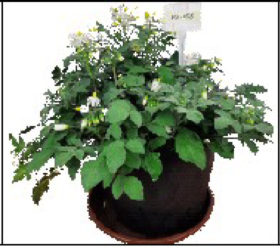	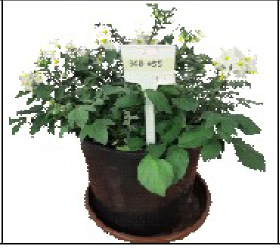	No tuber	BGB460	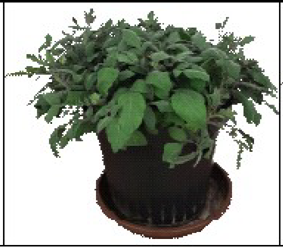	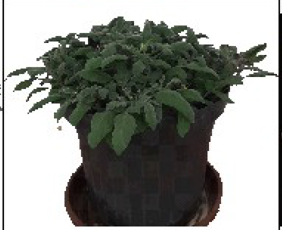	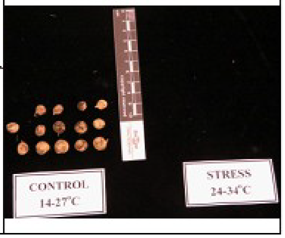

Figure S. 3: *Solanum commersonii* accessions after 35 days of heat stress under control and heat stress conditions, and tuber production under both conditions.

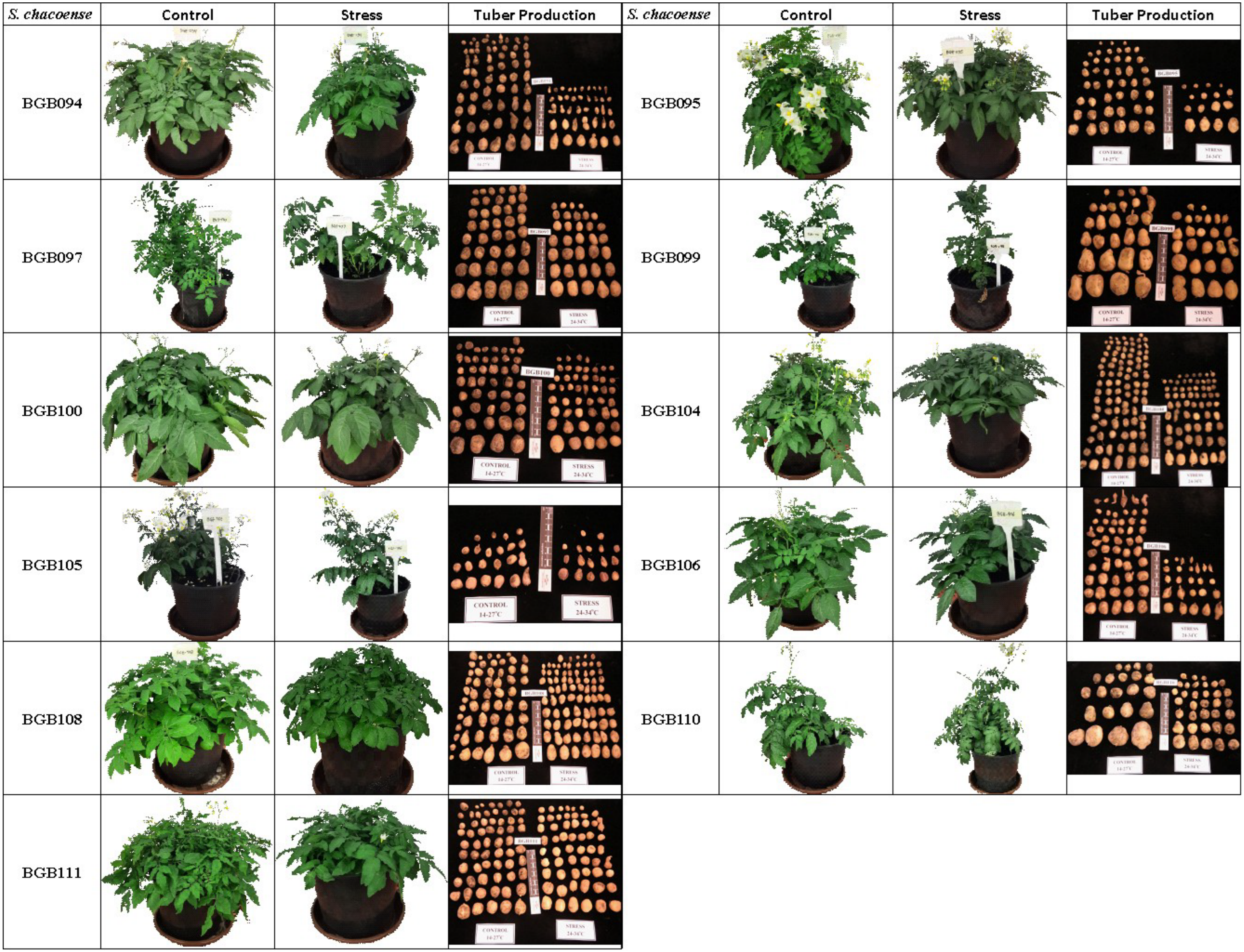

Figure S. 4: *Solanum chacoense* accessions after 35 days of heat stress under control and heat stress conditions, and tuber production under both conditions.

Table S. 1: Wild potato germplasm (Solanum) from Embrapa Potato Genebank used for screening under heat stress.

Sr. #	Local Code	Genesys #	Species	Origin
01.	BGB001	BRA 00167007-4	*S. commersonii*	*Brazil → RS → Ijuí*
02.	BGB011	BRA 00167027-2	*S. commersonii*	*Brazil → RS → Rio Grande*
03.	BGB048	BRA 00167400-1	*S. commersonii*	*Brazil → RS → São Sebastião do Caí*
04.	BGB055	BRA 00167407-6	*S. commersonii*	*Brazil → RS → Rio Grande*
05.	BGB068	BRA 00167420-9	*S. commersonii*	*Brazil → RS → São Gabriel*
06.	BGB077	BRA 00167429-0	*S. commersonii*	*Brazil → RS → Pejuçara*
07.	BGB094	BRA 00167446-4	*S. chacoense*	*Argentina → Córdoba*
08.	BGB095	BRA 00167447-2	*S. chacoense*	*Argentina → Córdoba*
09.	BGB097	BRA 00167449-8	*S. chacoense*	*Paraguay → Asunción*
10.	BGB099	BRA 00167451-4	*S. chacoense*	*Bolívia*
11.	BGB100	BRA 00167017-3	*S. chacoense*	*Argentina → Catamarca*
12.	BGB104	BRA 00167021-5	*S. chacoense*	*Argentina → Jujuy*
13.	BGB105	BRA 00167022-3	*S. chacoense*	*Argentina → Catamarca*
14.	BGB106	BRA 00167023-1	*S. chacoense*	*Argentina*
15.	BGB108	BRA 00167025-6	*S. chacoense*	*United Kingdom of England*
16.	BGB110	BRA 00167028-0	*S. chacoense*	*United Kingdom of England*
17.	BGB111	BRA 00167029-8	*S. chacoense*	*Paraguay → Asunción*
18.	BGB453	BRA 00183760-8	*S. commersonii*	*Brazil → RS → Herval*
19.	BGB460	BRA 00183766-5	*S. commersonii*	*Brazil → RS → Tapes*

Table S. 2: Analysis of variance for physiological and agronomical traits measured after 1, 15, and 35 days in 19 different wild genotypes of potato at 2 different temperature regimes.

	Source	DF	Pn	Gs	E	YII	NPQ	Fv/Fm	Chl-A	Chl-B	Cart
1DHS	Genotype (G)	18	24.02***	0.028***	3.85***	0.0037***	0.044***	0.011***	0.292	0.057	0.012
	Treatment (T)	1	3.05	0.0046	0.54	0.0053***	0.00045	0.038***	0.433	0.099	0.0019
	G × T	18	14.23**	0.0063*	0.74*	0.0027***	0.0096***	0.006***	0.488	0.088	0.015*
15DHS	Genotype (G)	18	50.33***	0.033***	6.53***	0.0082***	0.038***	0.0034***	0.424*	0.071	0.014*
	Treatment (T)	1	92.04***	0.120***	0.26	0.00077	0.0075**	0.0035***	0.514	0.006	0.032*
	G × T	18	15.99***	0.017***	2.46***	0.0014***	0.0093***	0.0027***	0.165	0.049	0.005
35DHS	Genotype (G)	18	11.48**	0.026***	2.56***	0.007***	0.037***	0.0015***	0.536	0.115	0.021
	Treatment (T)	1	110.02***	0.303***	167.21***	0.073***	0.199***	0.00046***	1.987*	0.524**	0.038
	G × T	18	6.46	0.010*	1.08	0.005**	0.011***	0.0012***	0.284	0.048	0.009

Table S. 3: Heat tolerance coefficient of potato wild relatives (Solanum) calculated from BLUP values predicted under control and heat stress conditions for fresh shoot weight (FSW), dry shoot weight (DSW), number of tubers (NT), tuber weight (TW), and dry matter (DM).

Genotype	Specie	FSW	DSW	NT	TW	DM
BGB001	*S. commersonii*	0.869	0.974	0.039	0.490	0.218
BGB011		0.807	0.962	0.353	0.098	0.822
BGB048		0.843	0.968	0.454	0.307	0.930
BGB055		0.874	0.973	0.039	0.490	0.218
BGB068		0.825	0.966	0.609	0.163	0.910
BGB077		0.836	0.958	0.217	0.222	0.870
BGB453		0.838	0.978	0.039	0.490	0.218
BGB460		0.853	0.967	0.218	0.180	0.590
BGB094	*S. chacoense*	0.812	0.962	0.763	0.592	0.885
BGB095		0.850	0.971	0.528	0.498	0.883
BGB097		0.762	0.954	0.781	0.769	0.888
BGB099		0.754	0.958	0.779	0.723	0.878
BGB100		0.843	0.961	0.887	0.614	0.892
BGB104		0.838	0.964	0.771	0.694	0.904
BGB105		0.857	0.976	0.671	0.266	0.914
BGB106		0.832	0.972	0.607	0.526	0.893
BGB108		0.813	0.969	1.093	0.761	0.861
BGB110		0.791	0.975	0.997	0.656	0.831
BGB111		0.825	0.965	0.860	0.766	0.896
